# Preoperative diagnosis of solitary pulmonary nodules with a novel hematological index model based on circulating tumor cells

**DOI:** 10.3389/fonc.2023.1150539

**Published:** 2023-05-03

**Authors:** Qiuxi Zhou, Qiao He, Ling Peng, Yecai Huang, Kexun Li, Kun Liu, Da Li, Jing Zhao, Kairong Sun, Aoshuang Li, Wenwu He

**Affiliations:** ^1^ Department of General Internal Medicine, Sichuan Cancer Hospital and Institute, Sichuan Cancer Center, Cancer Hospital Affiliated to University of Electronic Science and Technology of China, Chengdu, China; ^2^ Department of Clinical Laboratory, Sichuan Cancer Hospital and Institute, Sichuan Cancer Center, Cancer Hospital Affiliated to University of Electronic Science and Technology of China, Chengdu, China; ^3^ Department of Radiation Oncology, Sichuan Cancer Hospital and Institute, Sichuan Cancer Center, Cancer Hospital Affiliated to University of Electronic Science and Technology of China, Chengdu, China; ^4^ Department of Thoracic Surgery, Sichuan Cancer Hospital and Institute, Sichuan Cancer Center, Cancer Hospital Affiliated to University of Electronic Science and Technology of China, Chengdu, China; ^5^ Department of Respiratory Medicine, Sichuan Academy Medical Sciences, Sichuan Provincial People’s Hospital, Chengdu, China; ^6^ Department of Gastroenterology, Chengdu Third People’s Hospital, The Affiliated Hospital of Southwest Jiaotong University, Chengdu, China

**Keywords:** pulmonary nodules, diagnosis, biomarkers, hematological index model, nomogram

## Abstract

**Objective:**

Preoperative noninvasive diagnosis of the benign or malignant solitary pulmonary nodule (SPN) is still important and difficult for clinical decisions and treatment. This study aimed to assist in the preoperative diagnosis of benign or malignant SPN using blood biomarkers.

**Methods:**

A total of 286 patients were recruited for this study. The serum FR^+^CTC, TK1, TP, TPS, ALB, Pre-ALB, ProGRP, CYFRA21-1, NSE, CA50, CA199, and CA242 were detected and analyzed.

**Results:**

In the univariate analysis, age, FR^+^CTC, TK1, CA50, CA19.9, CA242, ProGRP, NSE, CYFRA21-1, and TPS showed the statistical significance of a correlation with malignant SPNs (*P <*0.05). The highest performing biomarker is FR^+^CTC (odd ratio [OR], 4.47; 95% CI: 2.57–7.89; *P <*0.001). The multivariate analysis identified that age (OR, 2.69; 95% CI: 1.34–5.59, *P* = 0.006), FR^+^CTC (OR, 6.26; 95% CI: 3.09–13.37, *P* <0.001), TK1 (OR, 4.82; 95% CI: 2.4–10.27*, P* <0.001), and NSE (OR, 2.06; 95% CI: 1.07–4.06, *P* = 0.033) are independent predictors. A prediction model based on age, FR^+^CTC, TK1, CA50, CA242, ProGRP, NSE, and TPS was developed and presented as a nomogram, with a sensitivity of 71.1% and a specificity of 81.3%, and the AUC was 0.826 (95% CI: 0.768–0.884).

**Conclusions:**

The novel prediction model based on FR^+^CTC showed much stronger performance than any single biomarker, and it can assist in predicting benign or malignant SPNs.

## Introduction

A solitary pulmonary nodule (SPN) is a single intraparenchymal lung lesion with a diameter of less than 3 cm. Most SPNs are benign nodules, such as pulmonary hamartoma and tuberculoma (1). The incidence of malignancies for SPNs ranged from 0.5% to 3.5%, mostly primary lung cancer (2). It depends on the age of patients, smoking status, history of cancer, nodule diameter, nodule volume, spiculated margins, and nodule location (3). The most common pathological types of malignant SPNs are adenocarcinoma and squamous cell carcinoma (4, 5). However, both nodules share similar imaging features, such as spiculated margins and lobulated structures (6, 7). The imaging diagnostics of lung cancer patients include morphological imaging modalities such as chest X-ray (CXR) and computed tomography (CT) and nuclear medicine procedures such as positron emission tomography (PET). Most of the pulmonary nodules smaller than 1 cm will not be visible on chest radiographs (8). Additionally, at least 95% of the nodules identified by computed tomography (CT) are benign (9). In clinical practice, differentiating malignant from benign nodules by conventional imaging alone has been challenging, with false positive and false negative rates up to 75% and 48%, respectively (10). Functional abnormalities can be found using PET before they appear morphologically on traditional imaging, and some studies have shown good diagnostic performance in SPN (11, 12). However, their performance is affected by the patient’s stratification. A meta-analysis comparing the diagnostic value of ^18^F-FDG-PET/CT versus CT observed no significant differences in sensitivity, specificity, PLR, NLR, DOR, and AUC (10). Serum biomarkers have many advantages over tissue-based detection, such as being non-invasive and easily repeatable. Nevertheless, they have low sensitivity in diagnosing malignancies yet high false-positive rates in benign tumors or infections (13). The utility of single serum biomarkers in SPN diagnosis is thus limited, and clinical guidelines generally recommend that combinations of serum biomarkers be used to improve detection efficiency (14). Though many prediction models have been developed, few are widely used in clinical practice (15, 16). It is, therefore, imperative to identify novel biomarkers and prediction models supporting the early diagnosis of non-small cell lung cancer (NSCLC).

Folate receptor alpha (FRα) is a glycoprotein that is anchored to the cell membrane of normal epithelial cells and highly expressed in a variety of tumor cells of epithelial origin, including lung, colorectal, ovarian, etc. (17–19). An FR-based CTC detection has been developed, and the related FR-positive CTC (FR^+^CTC) detection kit has been approved by the CFDA for clinical application. FR^+^CTCs have high sensitivity (73.2%–81.8%) and specificity (84.1%–93.2%) for the diagnosis of lung cancer (20, 21). FR^+^CTCs combined with common cancer biomarkers have been proven to improve diagnostic efficiency significantly in patients with NSCLC (20, 22). Xue et al. reported that FR^+^CTCs are reliable biomarkers that have a better performance than serum carcinoembryonic antigen (CEA), neuron−specific enolase (NSE), cytokeratin 19 fragments (CYFRA21−1), squamous cell carcinoma antigen (SCC), progastrin−releasing peptide (Pro-GRP), and heat shock protein 90−α (Hsp90α) in patients with small-sized nodules (23). FR^+^CTCs for the diagnosis of SPNs have been examined in a small prospective study (24). However, the utility of FR^+^CTC levels in combination with serum and tumor biomarkers to build a diagnostic model in NSCLC patients with SPNs was not reported.

In this study, we aimed to explore the expression of peripheral blood FR^+^CTCs, establish a diagnostic model based on FR^+^CTCs, and combine serum biomarkers in patients with SPNs. Furthermore, the study helps guide the clinical treatment strategies for pulmonary nodules.

## Methods

### Patients and data collection

**Figure 1:**
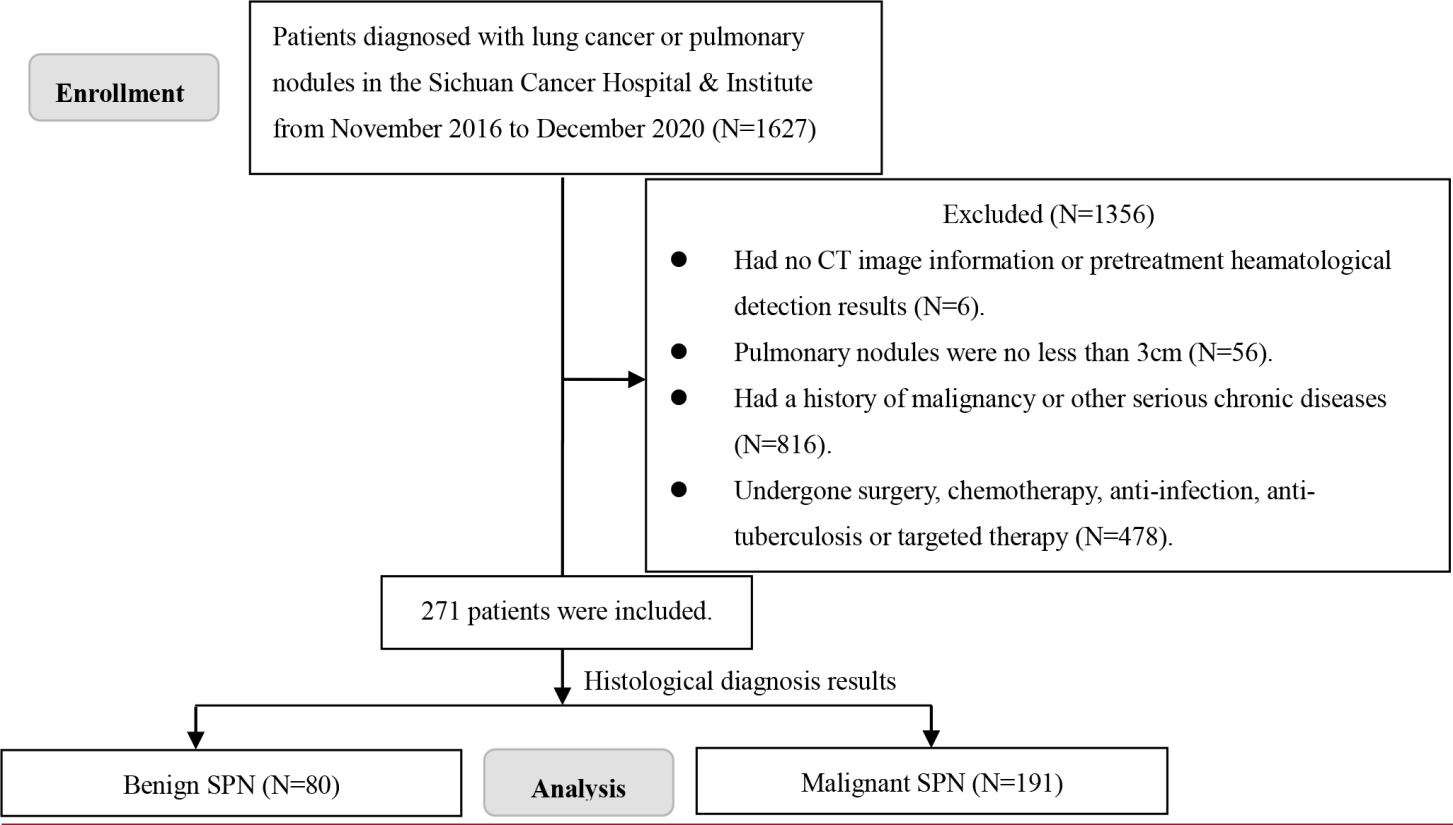
Screening flowchart of the participants.

A total of 1,627 patients diagnosed with lung cancer or pulmonary nodules at the Sichuan Cancer Hospital & Institute from November 2016 to December 2020 were analyzed retrospectively. Finally, 271 patients were included in this study (**Figure 1**) based on the inclusion and exclusion criteria as follows: (1) Patients with chest CT images indicated pulmonary nodules; (2) pulmonary nodules were less than 3 cm; and (3) pretreatment hematological detection, including folate receptor-positive circulating tumor cell (FR^+^CTC) level, thymidine kinase 1 (TK1), total protein (TP), albumin (ALB), pre-albumin (PALB), pro-gastrin-releasing peptide (ProGRP), recombinant cytokeratin fragment antigen 21-1 (CYFRA21-1), tissue polypeptide specific antigen (TPS), neuron-specific enolase (NSE), carbohydrate antigen 50 (CA50), carbohydrate antigen 199 (CA19.9), and carbohydrate antigen 242 (CA242) were available. Exclusion criteria: (1) patients had a history of malignancy or any other serious chronic diseases; and (2) patients underwent surgery, chemotherapy, anti-infection, anti-tuberculosis, or targeted therapy.

This study was approved by the Ethics Committee of Sichuan Cancer Hospital (No. SCCHEC-02-2017-042). All samples for hematological detection were collected from each patient before the initiation of treatment. Demographic characteristics were collected through the hospital information system (HIS). We present the following article in accordance with the Transparent Reporting of a multivariable prediction model for Individual Prognosis Or Diagnosis (TRIPOD) reporting guideline.

### FR^+^CTC extraction and quantification

After collection, whole blood samples for Transparent Reporting of a multivariable prediction model for Individual Prognosis Or Diagnosis FR^+^CTC detection were conducted within 24 h according to the manufacturer’s protocol for the CytoploRare Kit (Genosaber Biotech, Shanghai, China). At first, the erythrocytes were lysed with lysing buffer, and then leukocytes and macrophages were removed with anti-CD45 and anti-CD14, respectively. Secondly, the enriched samples were labeled with detection probes that contained conjugates of a tumor-specific ligand folic acid and a synthesized oligonucleotide. The oligonucleotide (5’-CTCAA CTGGT GTCGT GGAGT CGGCA ATTCA GTTGA GGGTT CTAA-3’) was used for subsequent PCR amplification.

Folate receptor-expressing cells were eluted and quantified by the ABI 7500 Real-Time PCR System (ThermoFisher, MA, USA) after washing out free conjugates. The primer sequences were as follows: forward primer 5’-TATGA TTATG AGGCA TGA-3’; reverse primer 5’-GGTGT CGTGG AGTCG-3’; and TaqMan probe 5’-FAM-CAGTT GAGGG TTC-MGB-3’. The quantitative analysis of FR-positive CTC was calculated through the amplification curve of the sample and standard reference.

### Detection of serum biomarkers

TK1 was detected by enhanced chemiluminescence dot blot assay (Sino-Swed Tong Kang Bio-Tech, Shenzhen, China). The serum TP, ALB, and pre-ALB were determined with a Clinical Laboratory Beckman Coulter AU5800. CA50 and CA242 were measured with the electrochemiluminescence immunoassay system CL-6000i (Mindray, China). In addition, the electrochemiluminescence immunoassay system LIAISON^®^ XL (Nanjing Tao Ze Bio-Technology, China) was also used to detect TPS and NSE. Moreover, ProGRP, CYFRA21-1, and CA199 were detected by the electrochemiluminescence immunoassay system Cobas E411 (Roche, Germany), respectively.

### Statistical analyses

At first, numerical data was applied to the normality test. Normally distributed data were shown as mean ± standard deviation (SD). Alternatively, other data were shown as medians (interquartile range, IQR). Student’s t-tests were used to analyze normally distributed data between groups. Also, non-normally distributed data were analyzed by the Mann–Whitney U test. Categorical data were presented as numbers (percentages) and compared using the Chi-square test. The clinical data and hematological biomarkers were used to construct a univariate logistic regression model and a multivariate logistic regression model for the whole cohort. The final multivariate logistic model was developed by stepwise regression to obtain the best result with the smallest Akaike information criterion (AIC) (25). A nomogram was drawn based on the multivariate logistic regression model. The validity of the nomogram was evaluated by the calibration curve and the Hosmer–Lemeshow goodness of fit test. The receiver operating characteristic (ROC) curve was used to evaluate the diagnostic value of hematological biomarkers based on the area under the curve (AUC). We define the maximum Youden Index as the optimal cutoff value. Statistical analysis was conducted using R software version 4.1.0 (The Free Software Foundation, Boston, MA, USA). The “pROC” and “ggplot2” packages were used to draw the ROC and calibration curves. The “generalhoslem” package was used to conduct the Hosmer–Lemeshow test. A two-sided P <0.05 was considered significant.

## Results

### Characteristics of malignant and benign SPNs

**Figure 2:**
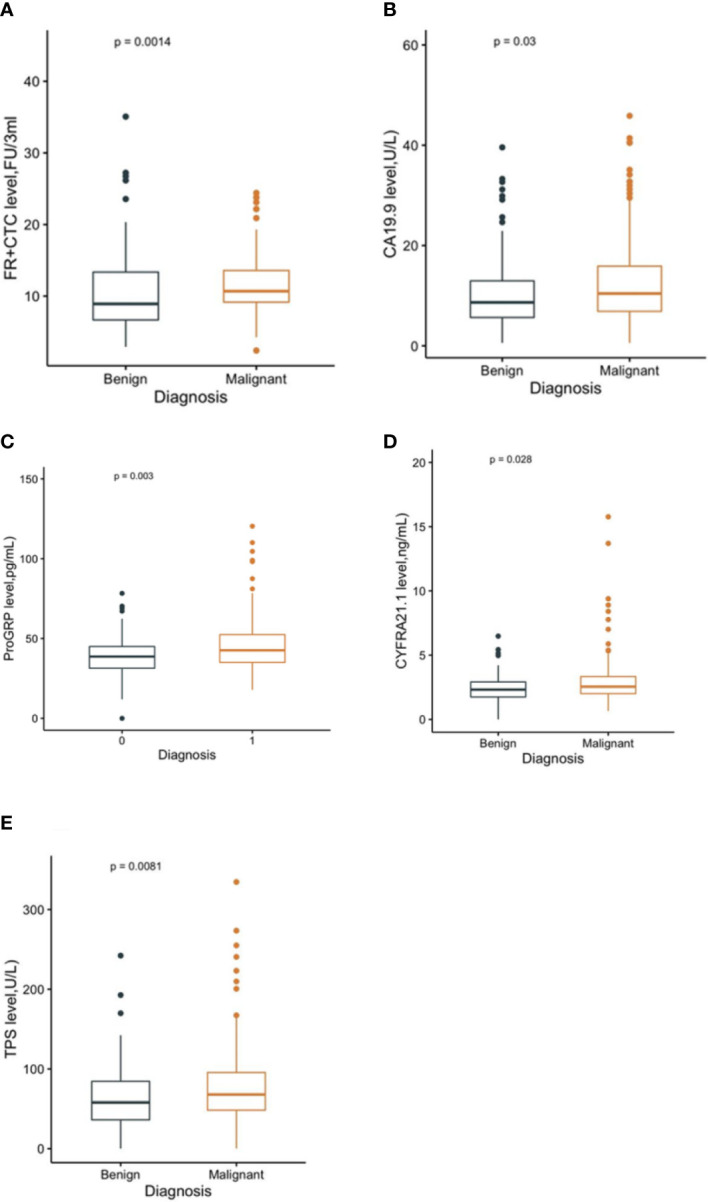
The level of FR^+^CTC **(A)**, CA19.9 **(B)**, ProGRP **(C)**, CYFRA21.1 **(D)**, and TPS **(E)** in malignant and benign groups, respectively.

In total, 191 malignant solitary pulmonary nodules (SPNs) and 80 benign SPN patients with pretreatment hematological biomarkers were included in this study (**Figure 1**). The mean age of the malignant and benign SPN groups was 59.24 ± 10.91 years old and 52.48 ± 9.51 years old, respectively. The median FR^+^CTC level in the malignant SPN group was 10.69 (95% CI: 9.16, 13.59), which was higher than that of the benign SPN group at 8.91 (95% CI: 6.68, 13.36) (*P* = 0.0014) (**Figure 2A**) (**Table 1**). CA19.9, ProGRP, CYFRA21.1, and TPS were significantly different between the malignant and benign groups (all *P* <0.05) (**Figures 2B–E**) (**Table 1**). The detailed information on the clinical characteristics and pretreatment hematological biomarkers of the patients is summarized in **Table 1**.

**Table 1:** Baseline characteristics of benign and malignant SPNs.

Characteristics	Overall (N = 271)	Benign SPN (N = 80)	Malignant SPN (N = 191)	P-value
Age (mean (SD), years)	57.24 (10.94)	52.48 (9.51)	59.24 (10.91)	<0.001
Sex (n, %)				
Female	140 (51.7)	44 (55.0)	96 (50.3)	0.563
Male	131 (48.3)	36 (45.0)	95 (49.7)	
FR^+^CTC (median [IQR], FU/3 ml)	10.36 [8.49, 13.52]	8.91 [6.68, 13.36]	10.69 [9.16, 13.59]	0.001*
TK1 (median [IQR], pM)	2.03 [1.46, 2.80]	1.82 [1.36, 2.66]	2.15 [1.50, 2.84]	0.073
TP (median [IQR], g/L)	64.30 [60.55, 67.25]	64.30 [61.25, 67.70]	64.30 [60.40, 67.20]	0.614
ALB (median [IQR], g/L)	39.10 [37.30, 41.70]	39.80 [37.00, 42.30]	39.10 [37.30, 41.60]	0.755
PALB (median [IQR], mg/L)	227.60 [202.90, 260.85]	229.10 [203.87, 262.45]	226.90 [202.70, 259.80]	0.968
CA50 (median [IQR], U/L)	5.89 [3.96, 8.64]	5.03 [3.47, 7.60]	6.19 [4.15, 8.95]	0.063
CA19.9 (median [IQR], U/L)	9.83 [6.46, 15.14]	8.66 [5.65, 12.95]	10.44 [6.90, 15.96]	0.026*
CA242 (median [IQR], U/L)	3.49 [2.05, 5.89]	3.38 [1.65, 5.49]	3.53 [2.24, 5.94]	0.14
ProGRP (median [IQR], pg/ml)	41.03 [34.22, 48.30]	38.67 [31.34, 44.99]	42.60 [34.99, 52.46]	0.003*
NSE (median [IQR], ng/ml)	9.99 [8.75, 11.49]	9.89 [9.15, 10.60]	10.09 [8.61, 11.70]	0.585
CYFRA21.1 (median [IQR], ng/ml)	2.46 [1.91, 3.20]	2.32 [1.75, 2.92]	2.56 [2.01, 3.34]	0.024*
TPS (median [IQR], U/L)	63.47 [45.85, 92.80]	57.92 [36.19, 84.44]	68.14 [48.30, 96.30]	0.007*

*indicates that it is statistically significant.

### Univariate and multivariate analysis of hematological biomarkers in distinguishing malignant SNPs

In the univariate analysis, sex, TP, ALB, and PALB were not significantly correlated with malignant SPNs (all *P* >0.05, **Table 2**). Age, FR^+^CTC, TK1, CA50, CA19.9, CA242, ProGRP, NSE, Cyfra21.1, and TPS showed statistical significance of correlation with malignant SPNs (all *P <*0.05, **Table 2**). The highest performing hematological biomarker is FR^+^CTC (odd ratio [OR], 3.44; 95% CI: 2.57–7.89; *P <0.001*) (**Table 2**). These factors, which showed significant results in the univariate analysis, were prepared for multivariate analysis. AIC was applied to variate selection, and age, FR^+^CTC, TK1, CA50, CA242, ProGRP, NSE, and TPS were included in the final multivariate prediction model. The formula of the prediction model was: (logit(p) = −3.09 + 0.99 ∗ Age + 1.83 ∗ CTC + 1.57 ∗ TK1 + 0.56 ∗ CA50 + 0.84 ∗ CA242 + 0.52 ∗ ProGRP + 0.72 ∗ NSE + 0.56 ∗ TPS). The multivariate analysis identified that age (OR, 2.69; 95% CI: 1.34–5.59; *P* = 0.006), FR^+^CTC (OR, 6.26; 95% CI: 3.09–13.37; *P* <0.001), TK1 (OR, 4.82; 95% CI: 2.40–10.27*; P* <0.001) and NSE (OR, 2.06; 95% CI: 1.07–4.06*; P* <0.001) are independent predictors (**Table 2**).

**Table 2:** Univariate and multivariate analysis of distinguishing malignant SNPs.

	Univariate analysis			Multivariate analysis		
Characteristic	HR	95% CI	P-value	HR	95% CI	P-value
Sex (male)	1.21	0.72–2.05	0.477	–	–	–
Age (≥60 years old)	3.44	1.93–6.4	<0.001*	2.69	1.34–5.59	0.006*
FR^+^CTC (FU/3 ml)	4.47	2.57–7.89	<0.001*	6.26	3.09–13.37	<0.001*
TP (g/L)	1.93	0.48–12.84	0.408	–	–	–
TK1 (pM)	2.41	1.42–4.16	0.001*	4.82	2.4–10.27	<0.001*
ALB (g/L)	0.69	0.41–1.17	0.166	–	–	–
PALB (mg/L)	0.74	0.44–1.26	0.263	–	–	–
CA50 (U/L)	2.26	1.33–3.87	0.003*	1.75	0.87–3.55	0.118
CA19.9 (U/L)	2.19	1.28–3.82	0.005*	–	–	–
CA242 (U/L)	2.56	1.35–4.85	0.004*	2.31	0.99–5.53	0.055
ProGRP (pg/ml)	2.37	1.39–4.1	0.002*	1.68	0.87–3.27	0.121
NSE (ng/ml)	2.11	1.21–3.79	0.01*	2.06	1.07–4.06	0.033*
CYFRA21.1 (ng/ml)	1.98	1.13–3.58	0.02*	–	–	–
TPS (U/L)	2.23	1.3–3.91	0.004*	1.74	0.92–3.36	0.093

*indicates that it is statistically significant.

### Diagnostic value of hematological biomarkers in distinguishing malignant SNPs

**Figure 3:**
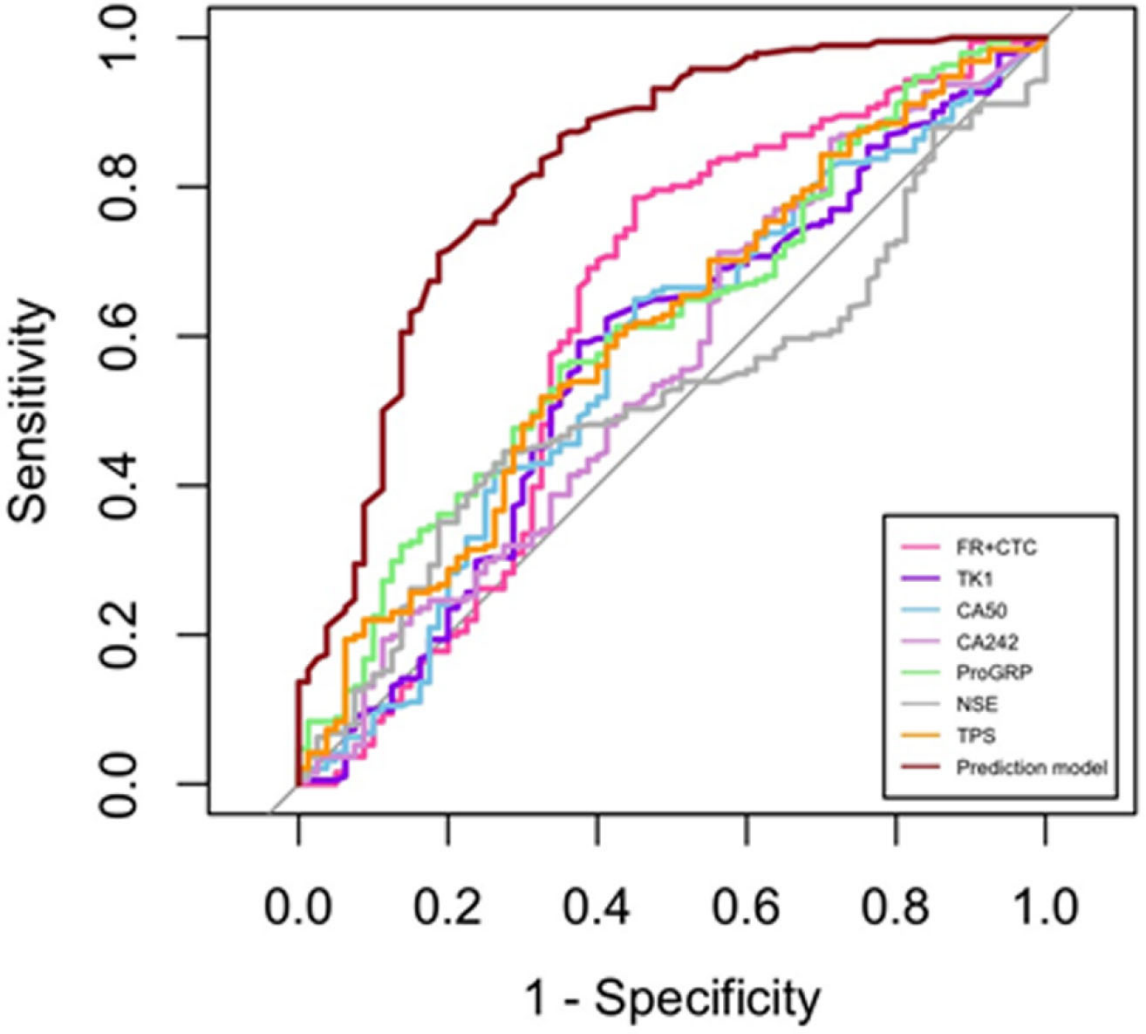
Receiver operating characteristic (ROC) curve-single marker and prediction model.

The ROC curve was used to further analyze the diagnostic value of pretreatment hematological biomarkers in distinguishing malignant SNPs (**Figure 3**). The optimal diagnostic cutoff values for FR^+^CTC, TK1, CA50, CA242, ProGRP, NSE, and TPS were 9.005 FU/3 ml, 1.965 pM, 5.24 U/L, 1.705 U/L, 41.085 pg/ml, 10.515 ng/ml, and 67.155 U/L, respectively. A single marker did not perform well in distinguishing between malignant and benign SNPs (all AUC <0.70) (**Table 3**). The multivariate prediction model, based on stepwise logistic regression and combined age, FR^+^CTC, TK1, CA50, CA242, ProGRP, NSE, and TPS, showed much stronger performance, with a sensitivity of 71.1% and specificity of 81.3%, and the AUC was 0.826 (95% CI: 0.768–0.884) (**Table 3**; **Figure 3**).

**Table 3:** The diagnosis values of hematological biomarkers.

Biomarker	Cutoff	Specificity	Sensitivity	AUC	95% CI	P-value(CTC reference)
FR^+^CTC	9.005	0.550	0.785	0.623	0.540–0.705	–
TP	74.45	0.975	0.047	0.481	0.405–0.556	0.012^#^
TK1	1.965	0.625	0.592	0.569	0.492–0.646	0.377
ALB	39.85	0.500	0.591	0.512	0.434–0.590	0.067
PALB	239.25	0.450	0.623	0.502	0.425–0.579	0.037^#^
CA50	5.24	0.550	0.649	0.572	0.495–0.649	0.382
CA19-9	10.375	0.675	0.513	0.586	0.51–0.661	0.527
CA242	1.705	0.288	0.864	0.557	0.479–0.634	0.262
ProGRP	41.085	0.650	0.560	0.614	0.542–0.687	0.873
NSE	10.515	0.725	0.445	0.521	0.449–0.593	0.075
CYFRA21-1	2.83	0.738	0.414	0.587	0.513–0.660	0.504
TPS	67.155	0.675	0.518	0.604	0.530–0.678	0.725
Prediction model^#^	–	0.813	0.711	0.826	0.768–0.884	<0.001

^#^Age + FR^+^CTC +T K1 + CA50 + CA242 + ProGRP + NSE + TPS.

### Nomogram development and validation

**Figure 4:**
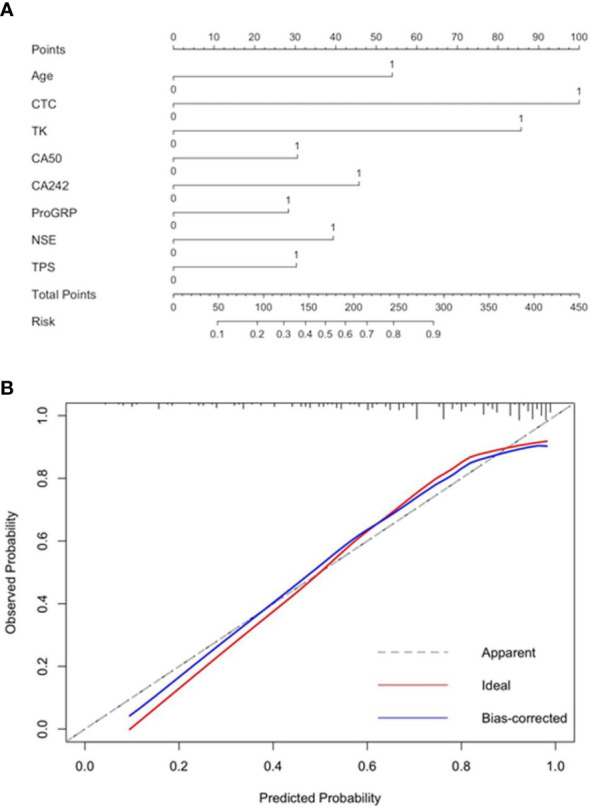
Nomogram **(A)** and the result of Hosmer–Lemeshow test for the model **(B)**.

The prediction model containing age, FR^+^CTC, TK1, CA50, CA242, ProGRP, NSE, and TPS was presented as a nomogram (**Figure 4A**). The Hosmer–Lemeshow test yielded significant goodness of fit (*P* = 0.04) (**Figure 4B**), and the C-index of the nomogram was 0.826 (**Table 3**).

## Discussion

The popularization of computed tomography (CT) increases the detection rate of pulmonary nodules. However, at least 95% of all identified pulmonary nodules are benign (9). Currently, the differentiation between benign and malignant SNPs smaller than 3 cm is still a major clinical challenge. A study by Laura et al. on ^18^F-FDG-PET/CT showed good diagnostic performance in SPN, reporting a sensitivity and specificity of 85.6% and 85.7%, respectively (12). However, they excluded indeterminate SPN patients. Moreover, in many infectious and inflammatory disorders with active macrophages, especially granulomatous diseases, FDG-PET may produce false positive results (10%–25%) (26). A review published in JAMA reveals that most of the detected benign nodules are granulomas or intrapulmonary lymph nodes (9), impacting the accuracy of PET imaging results. To improve the diagnostic accuracy, we detected the hematologic biomarkers of these patients and found that a single biomarker was poor at predicting the benign and malignant SNPs. Univariate and multivariate analyses were used to establish the first liquid biopsy model to predict benign and malignant SNPs. The novel predicting liquid biopsy model combined age, FR^+^CTC, TK1, CA50, CA242, ProGRP, NSE, and TPS, with a sensitivity of 71.1% and a specificity of 81.3%. It has excellent predictive value. In addition, the nomogram was generated from the predicting liquid biopsy model, which is more convenient for daily use by clinicians.

In a previous study, FR^+^CTC displayed the highest AUC compared with NSE, CEA, CA125, Cyfra21-1, and SCC Ag and could satisfactorily discriminate patients with NSCLC from controls, even in early-stage NSCLC (20). The results of our study are consistent with the previous study; the AUC of FR^+^CTC was higher than that of NSE and Cyfra21-1. In the study by Wang et al., FR^+^CTC showed the highest diagnostic efficiency in the diagnosis of lung cancer when compared with CEA, CYFRA21-1, and NSE. Notably, the combination of FR^+^CTC, NSE, CEA, and CYFRA21-1 could significantly improve diagnostic efficacy in differentiating patients with lung cancer from those with benign lung disease (27). Xue et al. reported that FR^+^CTC showed the highest AUC value among CEA, NSE, CYFRA21−1, SCC, ProGRP, and Hsp90α in the whole cohort and for participants with nodule sizes of ≤3 cm, the AUC, sensitivity, and specificity were 0.8063 (95% CI: 0.6769–0.9356), 80.00%, and 75.00%, respectively, which were lower than in the whole cohort (23). While in our study, all participants had a nodule size of ≤3 cm, however, the AUC, sensitivity, and specificity were lower in the above study. This difference may be caused by the small sample size. Recently, Zhou et al. found that the AUC of FR^+^CTC was the highest compared with CEA, CYFRA21-1, NSE, and SCC. The sensitivity and specificity for differentiating malignant from benign nodules were 78.6%–82.7% and 68.8%–78.4%, respectively (28). In our study, the prediction model was not developed based on significant factors in the results of multivariate analysis, while it was based on significant variables selected by AIC. In this way, enough variables were included in the model to avoid errors caused by the inclusion of variables only based on multivariate regression statistical differences. Our study found that the prediction model combined age, FR^+^CTC, TK1, CA50, CA242, ProGRP, NSE, and TPS had the best performance. However, multivariate analysis revealed that only older, higher FR^+^CTC levels, higher TK1 levels, and higher NSE are significant independent risk factors for malignant SPNs. By contrast, CA50, CA242, ProGRP, and TPS have been ignored. Among them, ProGRP was proven to be a novel biomarker in lung cancer (23). CA50, CA242, and TPS were proven to be novel biomarkers in lung cancer patients, although with a relatively lower AUC value (<0.7). In the previous study, CA50 and CA242 showed poor diagnostic efficacy for lung cancer screening with low AUC values. However, when combined with the other carbohydrate antigen (CA) biomarkers (CA125, CA15-3, CA19-9, and CA724), the AUC value was up to 0.776. Moreover, when coupled with CYFR21, CEA, NSE, and SCC, the AUC value was up to 0.884 (29). TPS is a specific fragment of keratin 18, which belongs to type I intermediate filaments found in epithelia. The TPS level significantly differed between the control and NSCLC groups, but multivariate analyses showed it was not an independent prognostic factor for advanced NSCLC (30). In the metastatic lung adenocarcinoma group, the TPS level is higher than in the non-metastatic group. However, it cannot predict the metastatic status because of the low AUC value (31). TK1 is strongly associated with DNA synthesis and cell proliferation and has demonstrated high diagnostic value in NSCLC. The serum levels of TK1 in NSCLC patients were higher than those of healthy individuals, and the AUC value was 0.667 (32), which is a promising biomarker for lung cancer.

Some limitations must be considered in our study. Firstly, this is a retrospective single-center study. A multicenter cohort study is warranted. Secondly, the sample size is not large enough. Therefore, it cannot represent the situation of all populations. Finally, the application value of this novel model is limited, and it is only suitable for the diagnosis of small pulmonary nodules. We will initiate a study on its relationship with prognosis after surgery.

## Conclusions

We established a preoperative prediction model with age and hematological indicators to improve the diagnostic workflow for small pulmonary nodules. In the meantime, we provide a nomogram that can be used for preoperative screening of early NSCLC patients and helps thoracic surgeons make a clinical decision.

## Data availability statement

The raw data supporting the conclusions of this article will be made available by the authors, without undue reservation.

## Ethics statement

The studies involving human participants were reviewed and approved by the Ethics Committee of Sichuan Cancer Hospital. The patients/participants provided their written informed consent to participate in this study.

## Author contributions

Conception and design: QZ and QH. Administrative support: WH and LP. Provision of study materials or patients: DL, JZ, and YH. Collection and assembly of data: KS, AL, KXL, and KL. Data analysis and interpretation: QZ and WH. Manuscript writing: QZ, QH, and WH. All authors contributed to the article and approved the submitted version.
